# The Transcription Factor c-Maf Promotes the Differentiation of Follicular Helper T Cells

**DOI:** 10.3389/fimmu.2017.00480

**Published:** 2017-04-27

**Authors:** Fabienne Andris, Sébastien Denanglaire, Maelle Anciaux, Mélanie Hercor, Hind Hussein, Oberdan Leo

**Affiliations:** ^1^Laboratoire d’Immunobiologie, Université Libre de Bruxelles, Brussels, Belgium

**Keywords:** CD4^+^ T lymphocytes, follicular helper T cells, humoral response, c-Maf, T cell differentiation

## Abstract

Follicular helper T cells (Tfh) have been identified as the primary cell subpopulation regulating B cell responses in germinal centers, thus supporting high-affinity antibody production. Among the transcription factors orchestrating Tfh cell differentiation and function, the role played by the proto-oncogene c-Maf remains poorly characterized. We report herein that selective loss of c-Maf expression in the T cell compartment results in defective development of Tfh cells in response to both antigen/adjuvant vaccinations and commensal intestinal bacteria. Accordingly, c-Maf expression in T cells was essential for the development and high-affinity antibody secretion in vaccinated animals. c-Maf was expressed early, concomitantly to BCL6, in Tfh cell precursors and found to regulate Tfh fate in a cell-autonomous fashion. Altogether, our findings reveal a novel, non-redundant, function for c-Maf in the differentiation of Tfh cells and the regulation of humoral immune responses to T-cell-dependent antigens.

## Introduction

Follicular helper T cells (Tfh) are key regulators of germinal center (GC) formation and T cell-dependent long-term humoral immunity. They provide crucial signals to B lymphocytes in GCs and guide high-affinity, isotype-switched, antibody responses, and memory B cell development (1).

Follicular helper T cells express the transcriptional repressor BCL6, considered as the critical master regulator of their development *in vivo* (2, 3) and are the major source of IL-21, which is necessary for IgG class-switch recombination and antibody-affinity maturation (4).

The differentiation of Tfh cells is considered as a multistage process starting in the T cell zone of secondary lymphoid organs. Here, T lymphocytes engage in cognate interaction and ICOS-ICOSL signaling with dendritic cells (DCs). These signals promote expression of CXCR5, allowing Th cells to relocalize at the T–B border zone where they receive additional signals from B cells (5, 6). This second wave of interactions further stabilizes Tfh cell fate—characterized by a high expression of BCL6 and surface markers such as CXCR5, PD1, ICOS—and results in the migration toward GCs and the delivery of optimal helper signals to B cells (5–7).

This stepwise differentiation pathway results from the sequential activation of a series of transcription factors regulating distinct phases of the Tfh developmental program. Within this intricate Tfh-associated transcriptional network, Ascl2 and BCL6 represent master regulators initiating Tfh cell development by inducing the expression of key Tfh-associated genes while inhibiting the expression of other, non-Tfh, helper cell subset signature genes (2, 3, 8, 9).

The transcription factor c-Maf, belonging to the AP-1 family of basic region/leucine zipper factor, is highly expressed by mature Tfh cells, and is thought to mainly regulate the expression of cytokines able to promote B cell proliferation and differentiation. Indeed, c-Maf is expressed downstream of Batf and ICOS signaling and has been shown to transactivate IL-4 and IL-21 promoters (10–12). In particular, Sahoo et al. recently reported that c-Maf promotes IL-4 secretion in Tfh cells through both direct binding to the CNS2 region in the *il4* locus and via induction of IRF4, thus revealing a distinct role of c-Maf in IL-4 secretion between Th2 and Tfh cell subsets (12).

Collectively, the available literature posits c-Maf as an important regulator of cytokine production in Tfh cells, thus acting at a later stage of the Tfh developmental program (1, 10, 12). To directly evaluate the putative role of c-Maf in the generation and regulation of Tfh activity, we have characterized the immune response of mice selectively lacking c-Maf expression in the T cell compartment. In contrast to our expectations, T cells lacking c-Maf expression failed to acquire expression of key Tfh markers (such as BCL6, CXCR5, and PD1), indicating an important, and non-redundant role for c-Maf in the initiation of Tfh cell development. Accordingly, mice lacking c-Maf in the T cell compartment displayed reduced secretion of high-affinity antibodies. Our data thus uncover a major and unsuspected role for c-Maf in regulating Tfh cell development and T-cell-dependent humoral responses.

## Materials and Methods

### Mice and Immunization

C57BL/6 mice were purchased from Envigo (Horst, The Netherlands). c-Maf-flox mice (13) were kindly provided by Dr. Carmen Birchmeier (Max Delbrück Center for Molecular Medicine, Berlin, Germany) and were back-crossed for nine generations to C57BL/6 in our animal facility before breeding with CD4-CRE mice (14), provided by Dr. Geert Van Loo (University of Gent, Gent, Belgium) to generate T-cell compartment-specific c-Maf-deficient mice (c-Maf^KO-T^ mice). CD3ε-KO mice were from EMMA (CDTA, Orleans, France).

All mice were used at 6–12 weeks of age.

Mice were immunized by injecting 10 μg keyhole limpet hemocyanin (KLH, Calbiochem) in foot pads (f.p.) along with Alum (1 mg/f.p., Thermo Fisher Scientific, Rockford, IL, USA) or IFA (sigma; 25 μL/f.p.) supplemented with LPS (*Escherichia coli* serotype 0111:B5, Calbiochem; 5 μg/f.p.). In some experiments, mice were immunized intra-peritoneally (i.p.) with 75 μg nitrophenyl-KLH (NP_25_-KLH, Biosearch Technologies, Novato, CA, USA) and 1 mg of Imject Alum. When indicated, mice were further boosted on day 14 by a second immunization with NP-KLH in saline.

### Differentiation of BMDCs

Bone marrow cells were collected from naive mice and grown for 8 days in RPMI supplemented with 10% FCS, 1% l-glutamine, 1% sodium pyruvate, 0.1% 2-ME, 50 μg/mL streptomycin, 50 IU/mL penicillin, and 20 ng/mL recombinant murine GM-CSF (provided by Pr. Kris Thielemans, Medical School of the Vrije Universiteit Brussel). At day 8, bone marrow-derived dendritic cells (BMDCs) were pulsed with 30 μg/mL KLH in the presence of 1 μg/mL LPS. At day 9, BMDCs were collected and injected in recipient mice (5 × 10^5^ cells/f.p.).

### Antibody Detection

Serum levels of NP-specific antibodies were determined by enzyme-linked immunosorbent assay (ELISA) according to standard procedures. Briefly, ELISA plates were coated with 2 μg/ml NP-BSA and incubated with serial dilutions of sera in duplicate wells. Bound antibodies were revealed using peroxidase-coupled anti-mouse isotype-specific rat monoclonal antibodies (Synabs sa, Louvain-la Neuve, Belgium) followed by the peroxidase substrate tetramethylbenzidine (Life Technologies). A solution of 2 N H_2_SO_4_ was used to quench the reaction, and optical densities were quantified at 450 nm. ODs were converted to units based on a standard curve made from a previously available immunized serum arbitrarily defined at 1,000 U ml^−1^.

The relative affinities of NP-immune sera were calculated by comparing their binding to differently haptenised carrier proteins (heavily haptenised NP_18_-BSA versus lightly haptenised NP_2_-BSA; Biosearch Technologies, Inc.), as described (15). The same serial dilutions of each serum sample were allowed to bind on NP_18_-BSA and NP_2_-BSA. The relative affinities of the anti-NP serum antibodies are expressed as a ratio of the serum volumes required to give the 50% of maximum binding on NP_18_-BSA divided by the volumes required for same binding on NP_^2^_-BSA (serum relative affinity = vol_50% binding_ on NP_^18^_-BSA/vol_50% binding_ on NP_2_-BSA).

### Flow Cytometry

Specific cell-surface staining was performed using a standard procedure with anti-CD4, anti-PD1, anti-IgD, anti-GL7 (eBioscience), and anti-CXCR5 mAbs (BD Biosciences).

Intracellular c-Maf, GATA-3, FoxP3, BCL6, Ki67 (Ab from BD Biosciences), and T-bet (eBioscience), staining was performed according to the manufacturer’s protocol (FoxP3 staining set protocol, eBioscience). Cells were analyzed by flow cytometry with a FACS Canto II (BD Biosciences) and analyzed with the FlowJo Software. Live cells were analyzed within a FSC-A/FSC-H gate to exclude cell doublets and triplets.

### Differentiation of Th Cells *In Vitro* and B-Cell Help Assay

CD62L^hi^CD4^+^ T cells and B cells were purified from naive animals by magnetic separation, as previously described (16, 17). The percentage of purified cell fractions in all experiments ranged between 90 and 98%, as estimated by flow cytometry (data not shown). Naive CD62L^hi^CD4^+^ T cells (5 × 10^5^ cells/well in 24-well plates) were activated for 48–72 h with plastic-coated anti-CD3 mAb (5 μg/mL) and soluble anti-CD28 mAb (1 μg/mL). Th1 cells were differentiated by the addition of recombinant IL-12 (10 ng/mL) and anti-IL-4 mAb (10 μg/mL), and Th2 polarization was initiated by the addition of IL-4 (10 ng/mL) and anti-interferon-γ (IFN-γ) mAb (10 μg/mL). Th17 and Tfh cells were differentiated in media containing IL-6 (20 ng/mL) and supplemented (Th17) or not (Tfh) with TGF-β (3 ng/mL), in the presence of anti-IL-4 and anti-IFN-γ mAbs.

Serial dilutions of *in vitro*-derived Tfh cells were co-cultured for 7 days with syngeneic naïve B cells (5 × 10^4^ cells/well) in the presence of anti-CD3 mAbs (500 ng/mL). T cells were irradiated (2,000 cGy) before the beginning of the coculture with B cells to prevent their outgrowth during the 7-day culture. IgG1 antibodies in the supernatants were determined by ELISA, using rat monoclonal anti-mouse isotype antibodies, as described (18) (Synabs, capture antibody loMG1.13; detection antibody loMK.1). Purified mouse IgG1 (BD Biosciences) was used as standard reference.

### Bone Marrow Chimeras

Recipient CD3ε-KO mice were lethally irradiated with 2 × 600 rad and reconstituted with a 1:1 mixture of bone marrow from congenic wild type CD45.1^+^ and c-Maf^KO-T^ (CD45.2^+^) mice (2 × 10^6^ cells of each). Chimeric mice were immunized with KLH/Alum 8 weeks after reconstitution.

### Peyer’s Patches Isolation

Peyer’s patches (PPs) were cut from the small intestine and incubated for 20 min at 37°C in Hank’s balanced salt solution (HBSS) containing 400 U/mL collagenase (Worthington, Lakewood, NJ, USA). PPs were next dissociated in HBSS/EDTA solution to generate a single-cell suspension for flow cytometry staining.

### Real-time Quantitative RT-PCR

RNA was extracted using the TRIzol method (Invitrogen) and reverse transcribed with Superscript II reverse transcriptase (Invitrogen) according to the manufacturer’s instructions. Quantitative real-time RT-PCR was performed using the SYBR Green Master mix kit (ThermoFisher). Primer sequences were as follow: RPL32 (F) ACATCGGTTATGGGAGCAAC; RPL32 (R) TCCAGCTCCTTGACATTGT; IL-4 (F) ATGCACGGAGATGGATGTG; IL-4 (R) AATATGCGAAGCACCTTGGA; IL-17A (F) ATCCCTCAAAGCTCAGCGTGTC; IL-17A (R) GGGTCTTCATTGCGGTGGAGAG; IL-21 (F) GCCAGATCGCCTCCTGATTA; IL-21 (R) CATGCTCACAGTGCCCCTTT; IFNγ (F) TGCCAAGTTTGAGGTCAACA; IFNγ (R) GAATCAGCAGCGACTCCTTT.

### Statistical Analysis

Differences between groups were analyzed with the Mann–Whitney test for two-tailed data. A *p*-value less than 0.05 was considered as significant.

## Results

### c-Maf Is Expressed in Early Tfh Cells during *In Vivo* Vaccination Settings and Is Required for Tfh Cell Development

**Figure 1:**
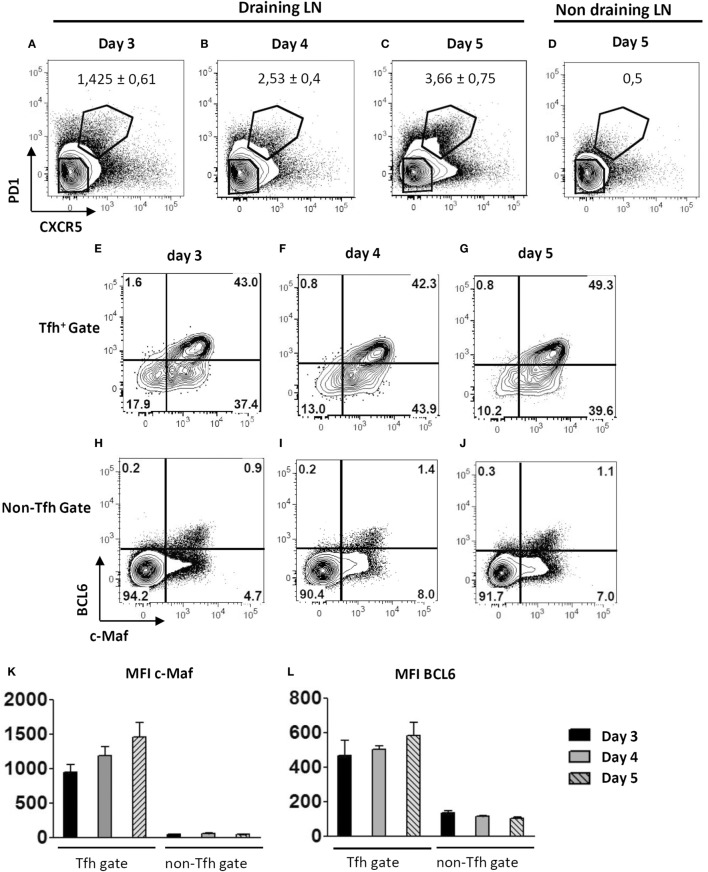
**Early expression of c-Maf during the course of follicular helper T cells (Tfh) cell differentiation**. WT mice were immunized by footpad injection of keyhole limpet hemocyanin in Alum. Draining lymph nodes were recovered on days 3–5 and analyzed for Tfh cell marker expression. Contour plots in **(A–D)** illustrate CXCR5 and PD1 staining profiles of CD4^+^ cells while contour plots in **(E–J)** represent BCL6 and c-Maf staining profiles of Tfh cells (CXCR5^+^ PD1^+^) and non-Tfh cells (CXCR5^−^ PD1^−^) gated as indicated in **(A–D)**; **(K,L)** histograms of c-Maf or BCL6 expression intensity (expressed as mfi, median fluorescence intensity) among Tfh and non-Tfh cell subsets. Results are representative of two independent experiments; histograms and numbers in **(A–D)** represent the mean ± SD of three individual mice.

Keyhole limpet hemocyanin/Alum vaccination promotes the *in vivo* differentiation of cells expressing key Tfh-associated markers including PD1, CXCR5, and BCL6 by 72 h, gradually increasing till day 5 (Figures 1A–D and data not shown). Kinetic analysis revealed that c-Maf was already expressed in the very few early Tfh found in the draining lymph nodes of day 3-vaccinated mice (Figure 1E). Although some c-Maf expression could be detected in the non-Tfh cell subset, higher levels of c-Maf were strongly associated with the Tfh cell compartment at any time points (Figures 1E–J). Moreover, c-Maf expression strongly correlated with BCL6 expression, therefore suggesting a time-coordinated expression pattern of these transcription factors throughout the Tfh cell differentiation process (Figures 1E–L).

**Figure 2:**
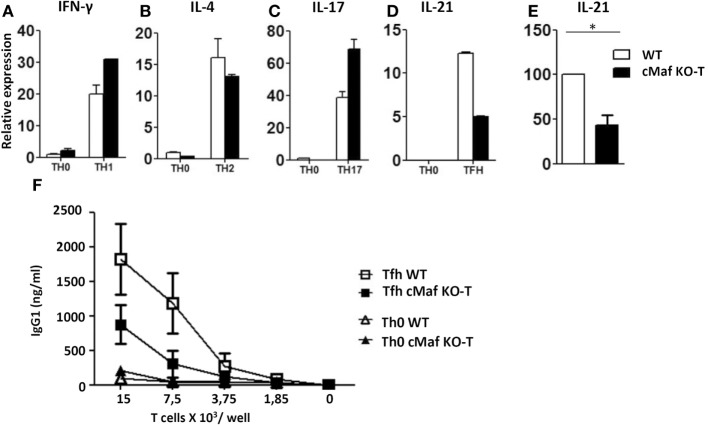
**c-Maf up-regulates IL-21 secretion and B-cell help capacity by follicular helper T cells (Tfh)-like cells *in vitro***. **(A–E)** Naïve T cells were purified from the spleen of C57BL/6 mice and stimulated for 2 days with anti-CD3/CD28 mAbs under Th0, Th1, Th2, Th17, or Tfh-like conditions. Gene expression relative to RPL32 mRNA of **(A)** interferon-γ, **(B)** IL-4, **(C)** IL-17, and **(D)** IL-21 was assessed by qRT-PCR. WT Th0 sample was set to 1. **(E)** Compilation of four independent experiments as in panel **(D)**: IL-21 expression in WT Tfh cells was set as 100. **(F)** Serial dilutions of Th0 and Tfh cells were incubated with purified B cells (5 × 10^5^ cells/well) and anti-CD3 mAbs (500 ng/ml). Culture supernatants were tested on day 7 for IgG1 content. Results are expressed as mean ± SD of duplicates **(A–D)** or four independent cultures **(F)** and are representative of at least two independent experiments. Differences between groups were analyzed with the Mann–Whitney test for two-tailed data. *p < 0.05.

To investigate the role of c-Maf in Tfh cell function and differentiation, we generated conditional KO mice in which *lox*P-flanked alleles of c-Maf (c-Maf^f/f^) are deleted by the Cre recombinase expressed from the CD4^+^ T-cell-specific *Cd4* promoter (c-Maf^f/f^-CD4^CRE^ mice, hereafter referred as c-Maf^KO-T^ mice). These mice showed normal T cell development *in vivo* (data not shown) and naïve c-Maf-KO Th cells underwent optimal Th1, Th2, or Th17 polarization *in vitro* (Figures 2A–C). Upon culture in Tfh-promoting conditions, c-Maf-KO T cells displayed a twofold reduction in IL-21 production (Figures 2D,E), in agreement with a role for c-Maf in regulating IL-21 production (11). Finally, c-Maf-deficient Tfh cells showed reduced ability to deliver B cell help for IgG1 secretion *in vitro* (Figure 2F).

**Figure 3:**
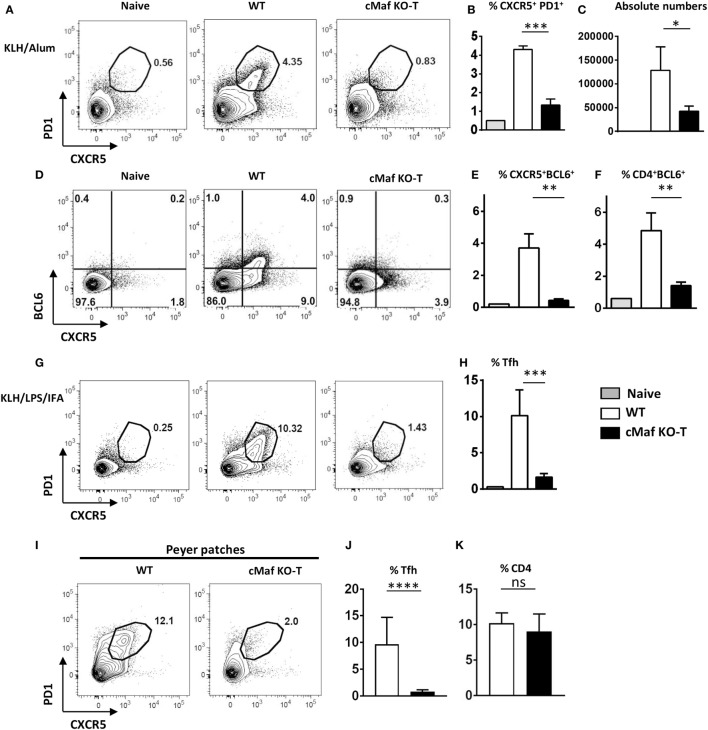
**c-Maf is required for follicular helper T cells (Tfh) cell development**. **(A–H)** c-Maf^fl/fl^ and c-Maf^KO-T^ mice were immunized with keyhole limpet hemocyanin (KLH) in Alum **(A–F)** or KLH + LPS in IFA **(G,H)**. Draining lymph nodes were analyzed on day 7 for Tfh cells (defined as CXCR5^+^ PD1^+^, CD4^+^ BCL6^+^, or CXCR5^+^ BCL6^+^) among viable CD4^+^ cells. **(A,D,G)** show representative contour plots; histograms **(B,C,E,F,H)** represent the percentages, and absolute numbers of Tfh cells, as indicated. **(I–K)** Tfh (CXCR5^+^ PD1^+^) and total CD4 T cell expression in Peyer’s patches of wild type and c-Maf^KO-T^ mice. Results represent the mean ± SD of four to five individual mice and are representative of at least three independent experiments **(B,C,E,F,H)** or are pooled from four independent experiments [*n* = 11–13 **(J,K)**]. Differences between groups were analyzed with the Mann–Whitney test for two-tailed data. *p < 0.05; **p < 0.01; ***p < 0.001; ****p < 0.0001.

To assess the role of c-Maf in Tfh cell development, we immunized wild type and c-Maf^KO-T^ mice with KLH/Alum or KLH/IFA/LPS. The loss of c-Maf strongly inhibited the differentiation of Tfh cells independently of the adjuvant formulation used (Figures 3A–H). The percentage and overall numbers of Tfh cells (assessed by either CXCR5^+^ PD1^+^, CD4^+^ BCL6^+^, or CXCR5^+^BCL6^+^ T cells) were significantly decreased in c-Maf^KO-T^ mice (Figures 3B,C,E,F,H).

**Figure 4:**
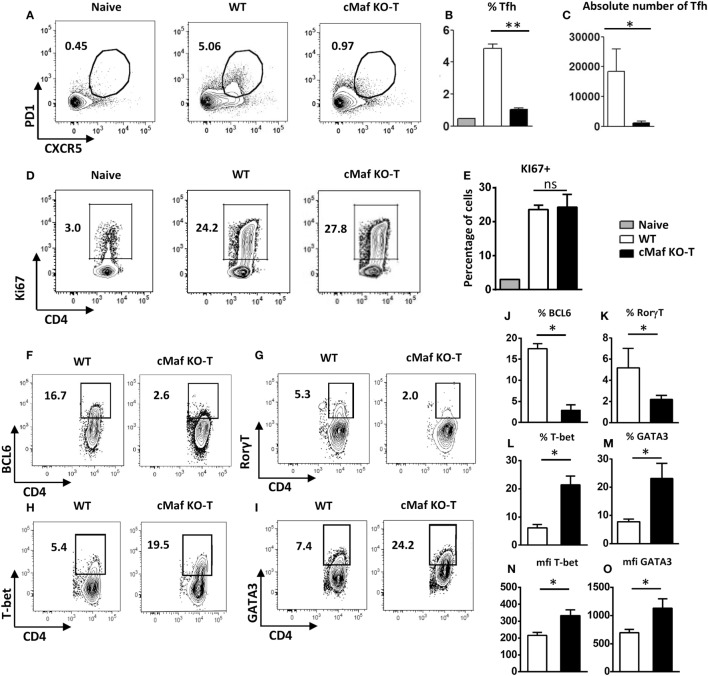
**c-Maf promotes follicular helper T cells (Tfh) cell differentiation and counteracts Th1 and Th2 cell development**. WT mice were immunized by footpad injection of 5 × 10^5^ LPS-stimulated, KLH-pulsed bone marrow-derived dendritic cells derived from C57BL/6 mice. Draining LN cells were recovered on day 7 and analyzed for Tfh cell expression **(A–C)** and Ki67 proliferation marker expression **(D,E)** among viable CD4^+^ T cells or BCL6, RORγt, T-bet, and GATA-3 expression among CD4^+^Ki67^+^ cell gate **(F–I)**. Histograms represent the percentage or absolute numbers of Tfh among viable CD4^+^ T cell gate **(B,C,E)**, percentages of cells among CD4^+^Ki67^+^ cell gate **(J–M)**, or transcription factor expression intensity [mfi **(N,O)**]. Differences between groups were analyzed with the Mann–Whitney test for two-tailed data. *p < 0.05; **p < 0.01; ***p < 0.001.

Inhibition of Tfh cell development *in vivo* was already evidenced at early time points following immunization (days 2 and 4, Figure S1 in Supplementary Material) and did not result from a general impaired Th cell response as judged by the presence of proliferating T lymphocytes (as assessed by expression of the Ki67 marker, data not shown and Figures 4D,E) in the corresponding lymph nodes.

To test whether these observations were also applicable to non-vaccinal-driven Tfh responses, we analyzed the Tfh response that spontaneously occurs in the intestinal PP of mice harboring commensal microbiota. The proportion of Tfh cells among CD4^+^ cell in PP was severely reduced in c-Maf^KO-T^ mice (Figures 3I–K), suggesting that c-Maf expression in T cells was also essential for normal homeostatic Tfh cell responses.

### c-Maf Promotes Tfh Cell Differentiation in a T-Cell Intrinsic Manner

To address the potential role of c-Maf in the regulation of Th cell responses *in vivo*, we immunized C57BL/6 mice with BMDCs loaded with KLH in the presence of LPS. We previously reported that this vaccination protocol leads to the coordinated upregulation of Tfh, Th1, Th2, and Th17 responses (19, 20). Analysis of the CD4^+^ T cell response further confirmed the defect in Tfh cell differentiation despite normal accumulation of CD4^+^Ki67^+^ cells in c-Maf^KO-T^ mice (Figures 4A–F,J). Notably, CD4^+^Ki67^+^ cells recovered from c-Maf^KO-T^ immunized mice expressed higher levels of T-bet or GATA-3, the major transcription factors associated with the Th1- or Th2-cell differentiation program, respectively (Figures 4H,I,L–O). As expected from the literature (21), c-Maf^KO-T^ mice failed to express optimal levels of RORγt following immunization (Figures 4G,K).

Overall, these observations point to a role for c-Maf in promoting Tfh and Th17 cell differentiation, while restraining the development of Th1 and Th2 lymphocytes upon antigen-stimulation *in vivo*.

**Figure 5:**
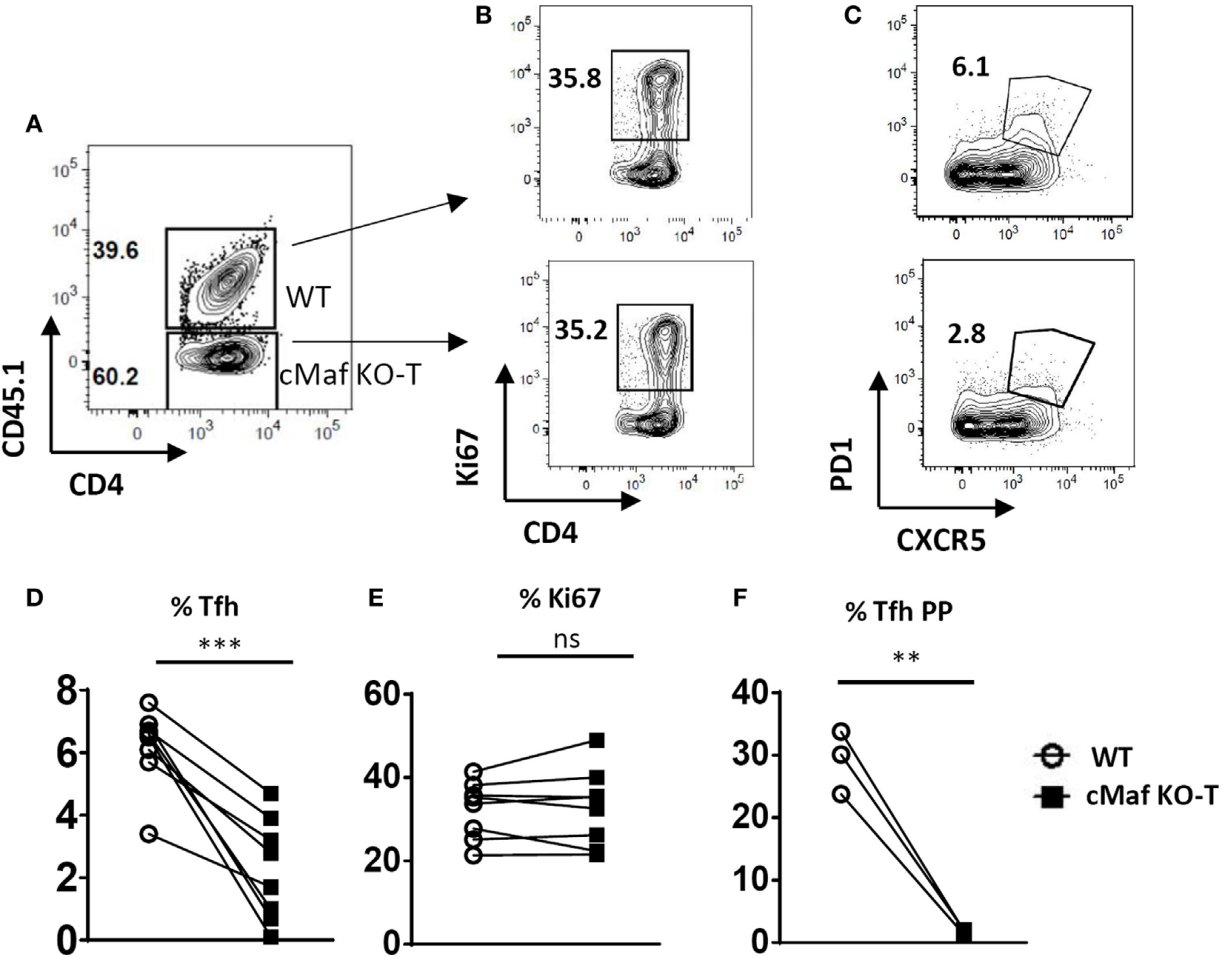
**c-Maf induces follicular helper T cells (Tfh) cell differentiation in a T-cell intrinsic manner**. Flow cytometry of CD4^+^ T cells in the spleen of chimeras generated with a mixture of WT (CD45.1) and c-Maf^KO-T^ (CD45.2) bone marrow cells, assessed 7 days after immunization with keyhole limpet hemocyanin/Alum in f.p. **(A)** gating strategy to identify WT and c-Maf-KO CD4^+^ T cells in the chimeric mice; **(B,C)** representative contour plots of activation and Tfh markers in both WT and c-Maf-KO CD4^+^ T cell subsets; **(D,E)** summary of Tfh and activated cell frequency from **(B,C)**; **(F)** summary of Tfh cell frequency in Peyer’s patches of chimeras. Each symbol represents individual mice. Results represent the mean ± SD of eight individual mice and are pooled from two independent experiments **(D,E)** or are representative of two independent experiments **(F)**. Paired Mann–Whitney test: **p < 0.01; ***p < 0.001.

We next generated chimeras by reconstituting irradiated CD3^−/−^ recipient mice with a 1:1 mixture of congenitally marked bone marrow cells from wild type (CD45.1^+^) and c-Maf^KO-T^ (CD45.2^+^) donor mice. Despite an equivalent proliferative response upon immunization (Figures 5A,B,E), cMaf-deficient CD4^+^ T cells displayed a reduced potential to acquire Tfh-features, both in the periphery (lymph nodes draining the immunization site, Figures 5C,D) and in the PP (Figure 5F), pointing to a cell-autonomous defect in the Tfh differentiation of c-Maf-deficient CD4^+^ T cells.

### c-Maf Expression in T Cells Is Required for Optimal Humoral Responses

**Figure 6:**
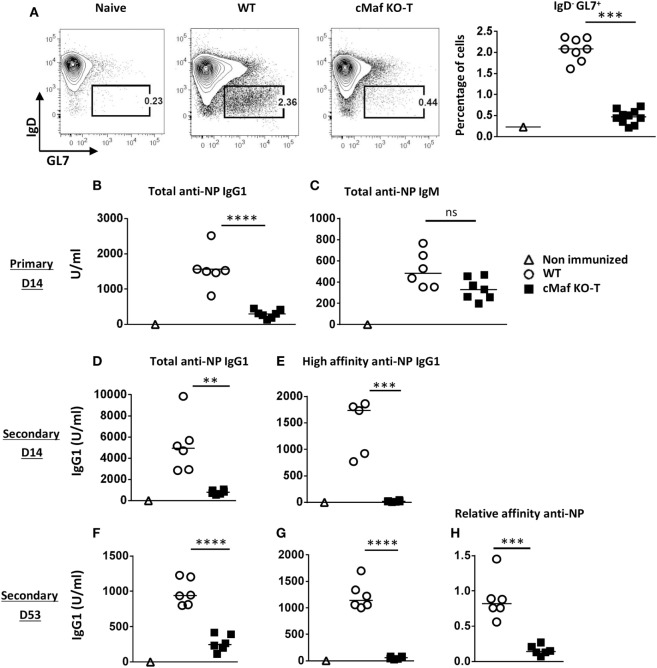
**c-Maf is required for high-affinity antibody response**. **(A)** Control and c-Maf^KO-T^ mice were immunized with keyhole limpet hemocyanin (KLH)/Alum in fp and draining lymph node were analyzed on day 7. Contour plots and histograms represent B_GC_ cells (defined as IgD^low^-GL7^+^, among CD19^+^ B cell gate). **(B–H)** Mice were immunized ip with NP-KLH in Alum. Sera were collected at the indicated timings and tested for total [fixation on NP_24_-BSA-coated enzyme-linked immunosorbent assay (ELISA) plates] anti-NP IgG1 **(B,D,F)** and IgM **(C)**. High-affinity IgG1 were detected on NP_4_-BSA-coated ELISA plates **(E,G)**. Relative affinity is expressed as the ratio of serum volume corresponding to 50% binding on NP_24_-BSA and NP_4_-BSA **(H)**. Each symbol represents individual mice. Results are pooled from two independent experiments **(A)** or are representative of three independent experiments **(B–H)**. Difference between groups was analyzed with the Mann–Whitney test for two-tailed data. **p < 0.01; ***p < 0.001; ****p < 0.0001.

We next tested whether c-Maf deficiency in the T cell compartment would affect the development of humoral responses. The loss of c-Maf led to a strongly reduced GC B cell response in the draining lymph nodes of immunized mice (Figure 6A). Accordingly, NP-KLH/Alum immunized c-Maf^KO-T^ mice produced lower anti-NP IgG1 antibodies titers at all time points (primary to late secondary response) considered, whereas anti-NP IgM levels remained non-significantly affected (Figures 6B–D,F). In particular, mice lacking c-Maf expression in T cells failed to secrete detectable levels of high-affinity NP-specific IgG1 antibodies (Figures 6E,G,H), in keeping with the concept that c-Maf-signaling in T cells promotes Tfh cell development, thereby controlling both isotype switch and affinity maturation processes.

## Discussion

While expression of cMaf has long been considered as a hallmark of Tfh cells, its precise role during Tfh cell development was unknown at the beginning of the present study. We demonstrate herein that c-Maf plays a major, non-redundant role in the differentiation of Tfh cells *in vivo*. c-Maf is expressed early during Tfh cell induction and mice harboring a T cell-specific conditional ablation of c-Maf are strongly impaired in their Tfh response to both antigen/adjuvant vaccinations and commensal intestinal bacteria.

By using retroviral ectopic expression of c-Maf or BCL6 in *in vitro*-derived human Tfh cells, Kroenke et al. first reported that c-Maf and BCL6 regulated distinct features of Tfh cell function, with BCL6 being critical for T cell positioning within the follicle and directing T–B interactions (through upregulation of CXCR5, CXCR4, CCR7, SAP, PD1, CD40L, ICOS, CXCL13) and c-Maf promoting and sustaining IL-21 secretion (10). The cooperation of c-Maf and BCL6/Ascl2 to achieve complete Tfh cell fate through orchestration of distinct sets of genes was further supported by studies indicating that c-Maf-bound genes hardly correlated with genes bound by BCL6 or Ascl2 (8, 9). Collectively, and in agreement with the well-known role of c-Maf in transactivating the IL-4 and IL-21 promoters (11, 12), these data suggested that c-Maf regulates Tfh cell function by fine-tuning cytokine production in Ascl2/BCL6-driven differentiated Tfh cells (1). Our data challenge this simple view of cMaf sustaining the Tfh program through cytokine, and in particular IL-21, production only. Indeed, c-Maf deficiency in the T cell compartment led to a virtually complete loss of Tfh cells, while lack of IL-21–IL-21R signaling appeared as largely compatible with Tfh cell development (22–24). Similarly, reduction in Tfh cell numbers was more severe in c-Maf^KO-T^ than in mice lacking STAT3 (the major IL-6/IL-21 signaling pathway in T cells) (20, 22, 25), thus suggesting that the defect observed in c-Maf^KO-T^ mice did not simply reflect a consequence of reduced IL-21 signaling in these cells.

Using a fetal liver transplant experiment, Bauquet et al. reported that loss of c-Maf resulted in markedly reduced numbers of Th17 and Tfh cells (26), suggesting that c-Maf might also regulate Tfh cell differentiation. However, the entire hematopoietic compartment (including T, B, and APCs) was defective for c-Maf in these chimeric mice, still leaving open the question of a direct intrinsic role of c-Maf in the Tfh cell differentiation program. In keeping with these conclusions, we provide evidence herein suggesting that c-Maf controls Tfh cell fate in a cell-autonomous fashion. Indeed, wild type Tfh cells failed to rescue Tfh differentiation of c-Maf-deficient T cells in chimeric mice, suggesting that soluble factors provided by wild type T cells cannot compensate for c-Maf loss during Tfh cell development.

As mentioned earlier, a first wave of pre-Tfh cell induction precedes the full Tfh cell differentiation process (characterized by stabilization of high levels of BCL6 expression) (5–7). Our *in vivo* studies reveal that c-Maf and BCL6 are co-expressed during this early step of Tfh cell development. Although previous studies and our own unpublished data have shown that over-expression of c-Maf does not directly induce BCL6 expression in Th cells (8, 10), we observe a severe defect in BCL6 expression in CD4^+^ Th cells, 1 week after immunization, in the absence of c-Maf. Collectively, these data thus suggest that promotion of the early wave of Tfh cell differentiation by c-Maf may facilitate further stabilization of BCL6 expression in fully committed Tfh cells.

*In vitro* studies confirmed that c-Maf expression was required for adequate IL-21 production (11). As previously documented, activation of naïve Th cells in the presence of IL-6 led to the differentiation of IL-21-producing Tfh-like cells able to induce B cell growth and antibody secretion (17, 18). Using the same *in vitro* assay, we observed a twofold reduction in IL-21 secretion by c-Maf-deficient Tfh-like cells, concomitantly with a reduced ability to deliver B cell help for antibody production in T–B co-culture assays. Thus, although failure to secrete adequate IL-21 levels may explain the reduced B cell help properties of c-Maf-deficient T cells *in vitro*, the strict requirement for c-Maf expression to achieve B cell help *in vivo* probably reflects additional roles for this transcription factor in driving Tfh development in a physiological setting.

The negative influence of c-Maf on Th2 responses may appear at odds with previous publications that led several groups to consider c-Maf as a prototypical Th2-transcription factor (27, 28). Notably, most of these studies were based on *in vitro* assays suggesting a positive role for c-Maf in regulating IL-4 promoter activity. However, Sato et al. later reported that c-Maf-transgenic memory T helper cells did not produce enhanced IL-4 levels when compared to WT cells, and that the expression of *Il4* was not reduced in immunodeficient mice reconstituted with c-Maf^−/−^ fetal liver transplants (29). In keeping with this last study, we observed an enhanced, rather than reduced, production of IL-4 by c-Maf-deficient T cells induced *in vivo* in response to antigen-pulsed DC vaccination. Similarly, c-Maf-deficient T cells displayed a marked Th1-like phenotype, further suggesting a possible role for c-Maf in controlling non-Tfh responses. These observations may suggest that similarly to BCL6, c-Maf may promote Tfh differentiation by limiting the acquisition of Th1/Th2 phenotypic traits. The identification of the precise mechanism whereby c-Maf may limit Th2 responses was beyond the scope of this study but might be related to limitation of GATA-3 expression in Th2 cells in our *in vivo* setting. Noteworthy, both GATA-3 and cMaf are involved in vertebrate lens development (30). Of interest, GATA-3 expression is strongly upregulated in c-Maf-deficient lens, a finding compatible with a negative role for c-Maf in regulating GATA-3 expression (31).

In conclusion, the fact that Tfh cell differentiation strongly relies on ICOSL/ICOS and IL-6/STAT3 signaling—two pathways known to induce expression of c-Maf (18, 32–34)—strongly supports our findings indicating an important role for c-Maf in driving Tfh cell differentiation. The present study thus adds c-Maf to a lengthy list of transcription factors (including BCL6, Ascl2, TCF-1, Batf, STAT3, NFAT, and IRF4) that have been shown to play a major, non-redundant role in Tfh cell development (1). The identification of the relative roles of these transcription factors in initiating and maintaining Tfh cell represents therefore an important challenge requiring further studies.

## Ethics Statement

The experiments were carried out in strict accordance with the relevant laws of the country and with institutional guidelines. We received specific approval for this study from the Université Libre de Bruxelles Institutional Animal Care and Use Committee (protocol numbers CEBEA-5, 31, and 92).

## Author Contributions

FA conceived and supervised the research program and experiments, acquired and analyzed data, and wrote the manuscript. SD, MA, MH, and HH performed the experiments and contributed to the analysis of the data. OL conceived the research program, provided financial support, and revised the manuscript.

## Conflict of Interest Statement

The authors declare no conflict of interest regarding the publication of this paper. The funders had no role in study design, data collection and analysis, decision to publish, or preparation of the manuscript.
