# Supplementary figures and images for: The Transcription Factor c-Maf Promotes the Differentiation of Follicular Helper T Cells
Source: Front Immunol. 2017 Apr 27;8:480. doi: 10.3389/fimmu.2017.00480 (PMC5406410; doi:10.3389/fimmu.2017.00480)

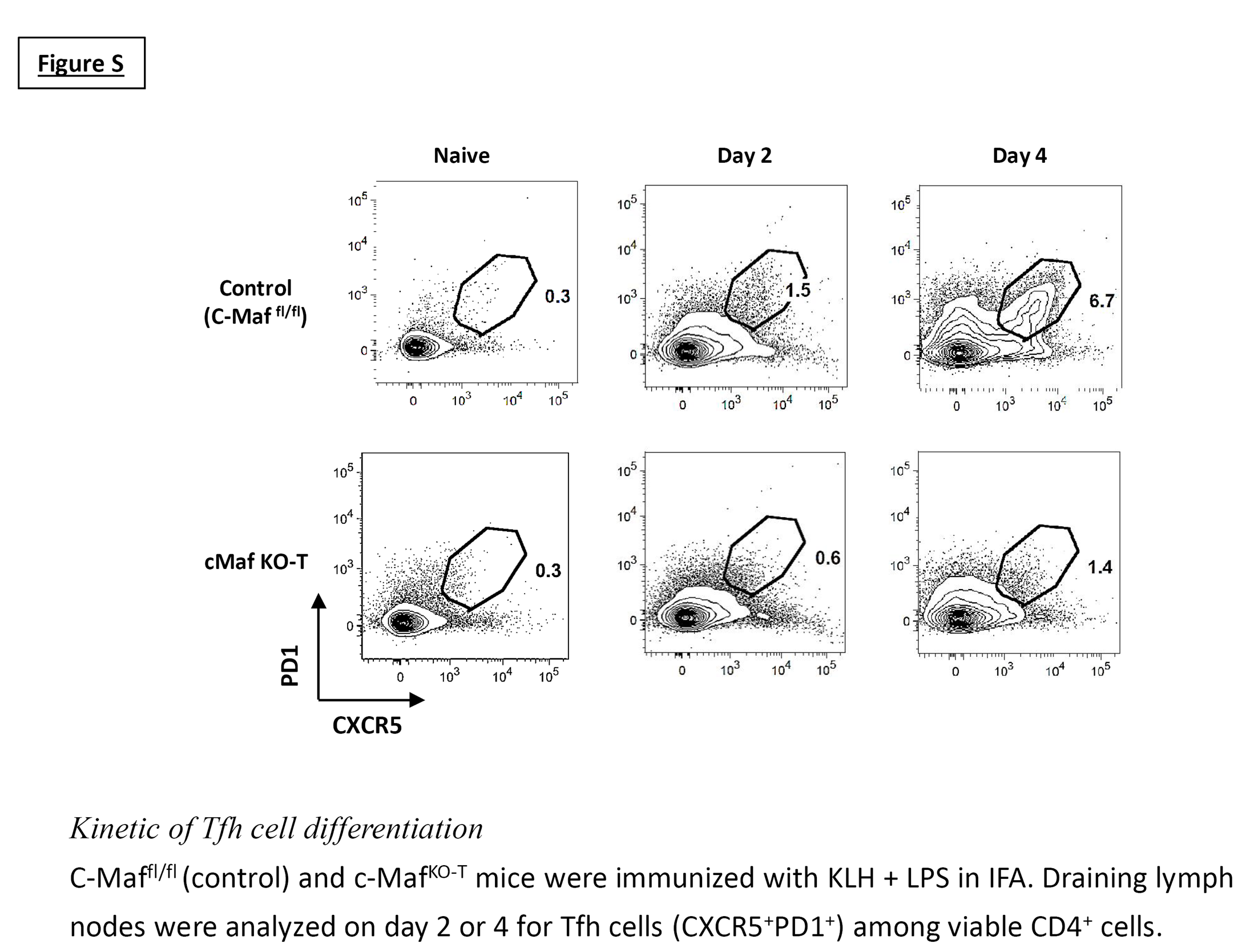

Supplement: Supplementary file 1 [file Image_1.tif]
